# A novel animal model of spontaneous epilepsy: Cdk5 knockout in pericyte-specific mice

**DOI:** 10.3389/fncel.2024.1474231

**Published:** 2024-10-16

**Authors:** Lin Lin, Xiaofei Hu, Weijun Hong, Tengwei Pan, Zhiren Wang, En Wang, Gang Wu

**Affiliations:** ^1^Department of Pharmacy, Taizhou Hospital of Zhejiang Province Affiliated to Wenzhou Medical University, Linhai, Zhejiang, China; ^2^School of Pharmacy, Wenzhou Medical University, Wenzhou, Zhejiang, China; ^3^Department of Neurology, Taizhou Hospital of Zhejiang Province Affiliated to Wenzhou Medical University, Linhai, Zhejiang, China; ^4^Taizhou Key Laboratory of Pharmaceuticals Therapy and Translation Research, Linhai, Zhejiang, China

**Keywords:** animal model, epilepsy, Cdk5, pericyte, AQP4

## Abstract

Changes in neurovascular unit components and their interactions play a crucial role in epileptogenesis and the pathological process of epilepsy. Currently, there is a lack of animal models that can accurately reflect the etiological impact of cerebrovascular lesions on epilepsy. In this study, we constructed cyclin-dependent kinase 5 conditional knockout mice in Cspg4 (pericyte marker)-positive cells using the Cre-LoxP system. The results revealed that this strain of mice exhibited significant seizure behaviors and epileptiform brain waves, loss of hippocampal and amygdala neurons, astrogliosis, decreased pericyte coverage, and reduced AQP4 polar distribution. Herein, we have developed a novel mouse model of spontaneous epilepsy, providing a critical animal model for studying the involvement of neurovascular unit factors in the development and progression of epilepsy.

## 1 Introduction

Despite regular antiepileptic drug treatment, about 25% of patients suffer from medically intractable epilepsy (Krishnamurthy, 2016). Further exploration of the pathogenesis of epilepsy is needed to uncover new drug targets. Current information on the etiology and pathological changes of epilepsy suggests that it is closely linked to cerebrovascular function, and changes in the components of the neurovascular unit and their interactions play an important role in epileptogenesis and pathological processes (Reiss et al., 2023; Dadas and Janigro, 2018). However, there is a lack of animal models that accurately reflect the etiological effects of cerebrovascular lesions on epilepsy.

Recent studies have shown that the redistribution of vascular pericytes is involved in the occurrence and development of epilepsy (Garbelli et al., 2015). Cyclin-dependent kinase 5 (Cdk5) is primarily involved in the migration, proliferation, and secretion of non-neuronal cells (Liebl et al., 2010). Using the Cre-LoxP system, our team constructed Cdk5 conditional knockout mice with Cspg4 (pericyte marker)-positive cells and found that these mice exhibited significant seizures and epileptiform brain waves at 24–32 weeks of age.

## 2 Methods

### 2.1 Animals

*Cspg4Cre* mice (purchased from The Jackson Laboratory, stock no. 00Cspg4) and *Cdk5^f/f^* mice (purchased from The Jackson Laboratory, stock no. 014156) were crossed to obtain *Cspg4Cre;Cdk5^f/n^* mice. These mice were then crossed to produce *Cspg4Cre;Cdk5^f/f^* mice, with *Cspg4Cre*, *Cdk5^f/n^*, or *Cdk5^f/f^* littermates used as controls. Mouse tail samples were collected, and DNA was extracted for gene identification via PCR-agarose gel electrophoresis. Primer sequences for Cdk5 were CAGTTTCTAGCACCCAACTGATGA (Forward) and GCTGTCCTGGAACTCCATCTATAGA (Reverse); primer sequences for Cre were CAGCATTGCTGTCACTTGGTC (Forward) and ATTTGCCTGCATTACCGGTCG (Reverse). All experimental protocols and animal handling procedures were approved by the Committee for Animal Experiments at Taizhou Hospital of Zhejiang Province in China (Approval No. tzy-2019008).

### 2.2 Video and EEG detection

At around 6 weeks of age, seizures were monitored and recorded via video surveillance for later playback analysis. Recordings were made 24 h a day until the first seizure of the last mouse was observed. Seizures were evaluated following Racine’s scale (Racine, 1972): stage 1 involves mouth and facial muscle contractions; stage 2 includes head nodding; stage 3 is characterized by unilateral or bilateral forelimb clonus; stage 4 involves rearing on the hindlimbs with forelimb clonus; and stage 5 is marked by rearing and falling with forelimb clonus. Due to the difficulty in observing subtle seizures, such as nodding and facial twitching in mice, the experiment primarily focused on seizures of grade 3 and above.

*Cspg4Cre;Cdk5^f/f^* mice were bred, and electrodes were implanted in their cortex and amygdala at the age of 14–15 weeks. Cortical EEG electrode screws (0.8 mm in diameter and 2 mm in length) were installed in the middle of the anterior and posterior fontanel, 1.5 mm from the sagittal suture. Amygdala electrodes (A-M system, 0.005”, Cat No. 791500) were implanted with coordinates of AP −1.8 mm, DL +3.1 mm, and DV −4.5 mm. Occipital screws were used as the reference, and anterior olfactory bulb screws were used as the ground electrode. EEG recordings were conducted with Brainvision Record after 7 days of recovery. EEG analysis was performed using Brainvision Analyze 2.0 with filters set to LowPass at 1 Hz, HighPass at 100 Hz, and Notch at 50 Hz. The EEG criteria for seizures included a wave frequency greater than 5 Hz, a wave amplitude more than twice the baseline, and a progressive temporal increase in both frequency and amplitude lasting longer than 10 s (Pun et al., 2012).

### 2.3 Tissue immunofluorescence test

After anesthesia, the mice were perfused with PBS and 4% paraformaldehyde solution, and the brains were removed. After fixation in 4% paraformaldehyde solution for 6 h, the brains were placed in 30% sucrose in PBS and then sectioned at 20 μm using a cryostat. Prior to staining, the slices were washed three times with PBS, treated with 1% Triton-X100 for 15 min, blocked with donkey serum for 1 h, and incubated at 4°C for 2 days with diluted GFAP antibody (Abcam, ab7260), NeuN antibody (MilliporeSigma, ABN78), PDGFRβ antibody (Abcam, ab32570), or Glut1 antibody (Abcam, ab40084). After washing three times with PBS, fluorescent secondary antibodies were added and incubated in the dark for 4 h. Following another three washes with PBS, 5 μM DAPI solution was added and stained for 15 min. The samples were then washed three times with PBS, mounted with an anti-quencher reagent, and imaged using confocal laser scanning microscopy.

### 2.4 Pericyte vascular coverage and AQP4 polar distribution assay

Image analysis was performed using ImageJ (NIH, USA) software. Pericyte vascular coverage was assessed by dividing the PDGFRβ-positive area by the Glut1-positive area in the perivascular zone. All images were set at uniform high and low stringency thresholds. A low stringent threshold defines the overall region of AQP4 immunoreactivity, while a high stringent threshold defines AQP4 signals confined to the perivascular endfeet. The ratio of fluorescence intensity at high and low stringent thresholds is the AQP4 polarity index; a larger index indicates a higher polarity distribution (Wang et al., 2012).

### 3 Results

### 3.1 Video-EEG monitoring of pericyte conditional knockout Cdk5 transgenic mice

**FIGURE 1:**
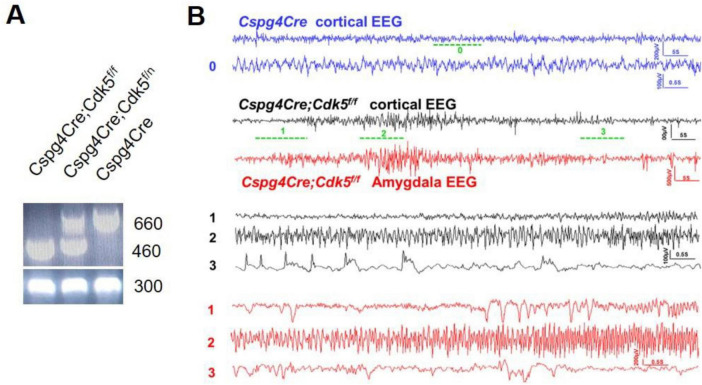
Gene identification and video-EEG recording of pericyte conditional knockout Cdk5 mice. **(A)** Representative results of gene identification using the agarose gel electrophoresis method. **(B)** Representative EEG recordings from the cortex and amygdala of control and mice with epilepsy. Mice *n* = 3.

Pericyte conditional knockout Cdk5 transgenic *Cspg4Cre;Cdk5^f/f^* mice were constructed using the Cre-LoxP method and identified by PCR and agarose gel electrophoresis (Figure 1A). Sixteen *Cspg4Cre;Cdk5^f/f^* mice and eight *Cspg4Cre* mice were video-monitored. Through video playback monitoring, we observed that both sexes of *Cspg4Cre;Cdk5^f/f^* mice began to exhibit seizures of grade 3 or higher at approximately 83 ± 10 days of age. Initially, we observed forelimb clonus, which gradually progressed to rearing on the hindlimbs (stage 4) and eventually to rearing and falling with forelimb clonus (stage 5) as the epilepsy advanced. Seizures were not observed in control groups. EEG monitoring detected epileptic waves in the cortex and amygdala of 16-week-old transgenic animals (Figure 1B), which occurred spontaneously without stimulation. However, the behavioral performance of 16-week-old transgenic animals in the open field test, elevated O-maze, light-dark box test, tail suspension test, and auditory startle/prepulse inhibition (PPI) test did not differ from controls (data not shown). Thus, pericyte conditional knockout Cdk5 transgenic mice were identified as spontaneously epileptic.

### 3.2 Neuronal loss and gliosis in pericyte knockout Cdk5 transgenic mice

**FIGURE 2:**
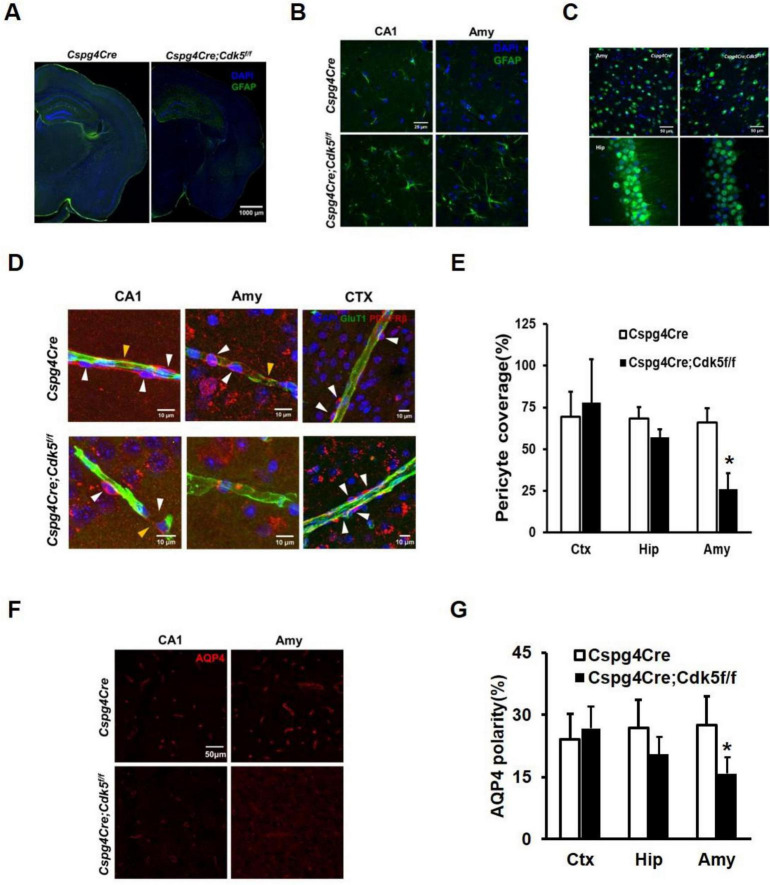
Immunofluorescence staining of astrocytes and neurons, vascular coverage of pericytes, and polar distribution of AQP4 in conditional pericyte knockout Cdk5 mice. **(A)** Illustration of GFAP immunofluorescence in brain slices. Green represents GFAP and blue indicates DAPI, with a scale of 1,000 μm, *n* = 4. To avoid overexposure, the Gain values were lowered in *Cspg4Cre;Cdk5^f/f^* group. **(B)** Magnified images of GFAP staining in the amygdala and hippocampus, with a scale of 25 μm, *n* = 4. **(C)** NeuN immunofluorescence showing neuronal loss in the amygdala and hippocampus. NeuN is shown in green and DAPI in blue, with a scale of 50 μm, *n* = 4. **(D)** Representative images of PDGFRβ-positive pericytes in the hippocampal CA1 region, amygdala (Amy), and cortical (CTX) areas, along with Glut1-positive endothelial cells, in *Cspg4Cre;Cdk5^f/f^* and control animals. White arrows indicate pericyte cell bodies, and yellow arrows indicate pericyte membranes. **(E)** Statistical plots of pericyte vascular coverage in *Cspg4Cre;Cdk5^f/f^* and control animals. Six animals per group, with five vessels analyzed per animal, *p < 0.01. Independent sample *T*-test statistics were used. AQP4 immunofluorescence in the CA1 region of the hippocampus and amygdala **(F)** and statistical analysis of AQP4 polar distribution **(G)** in *Cspg4Cre;Cdk5^f/f^* and control animals. Six animals per group, *p < 0.01. Independent sample *T*-test statistics were used.

Astrogliosis and neuronal loss are evident in the brains of mice with epilepsy (Seifert et al., 2010; Rho and Boison, 2022; Seifert and Steinhäuser, 2013). Even with reduced Gain values, significant astrogliosis was still evident in the hippocampus and amygdala of the *Cspg4Cre;Cdk5^f/f^* epileptic mice compared to controls (Figures 2A, B). Additionally, the number of neurons was decreased in *Cspg4Cre;Cdk5^f/f^* group (Figure 2C).

### 3.3 Reduced pericyte vascular coverage and AQP4 polar distribution in pericyte knockout Cdk5 transgenic mice

We further investigated the underlying mechanism of spontaneous epilepsy in pericyte knockout Cdk5 mice. Changes in the dynamic distribution of pericytes around blood vessels are implicated in the progression of both acute and chronic epilepsy (Arango-Lievano et al., 2018). Using immunofluorescence assays and ImageJ software, we observed reduced vascular coverage of pericytes in the amygdala of *Cspg4Cre;Cdk5^f/f^* mice (Figures 2D, E). Additionally, previous studies have indicated that AQP4 is predominantly distributed in the perivascular regions of blood vessels (Gundersen et al., 2014; Hoddevik et al., 2017), and its polar distribution in astrocytes is diminished in specimens from epileptic patients (Eid et al., 2005). Therefore, we examined the polar distribution of AQP4 and found a significant reduction in the amygdala of *Cspg4Cre;Cdk5^f/f^* mice. In the hippocampus, there was a tendency toward decreased pericyte vascular coverage and AQP4 polar distribution, although the decrease was not statistically significant (Figures 2F, G).

## 4 Discussion

We generated a mouse model of spontaneous epilepsy by knocking out the Cdk5 gene in pericytes. This model exhibits a distinct epileptic behavioral phenotype, epileptic EEG waves, and histopathological changes including gliosis and neuronal loss.

The mechanism of epilepsy in this strain of mice may be associated with reduced pericyte vascular coverage. Cdk5 primarily regulates the migration, proliferation, and secretion of non-neuronal cells (Liebl et al., 2010). Pericytes play a crucial role in maintaining the permeability of the blood–brain barrier (BBB), and a reduction in pericyte coverage disrupts BBB integrity, particularly in brain regions like the cortex and hippocampus (Armulik et al., 2010; Villaseñor et al., 2017). Increased BBB permeability results in elevated brain levels of mIgG, leading to neuronal toxicity (Armulik et al., 2010). Moreover, heightened BBB permeability can induce inflammatory responses and alter neuronal electrophysiological activity, contributing to the onset of epileptic behaviors (Liu et al., 2020). The influx of albumin following BBB breakdown activates TGF-β receptor type 2 in astrocytes, inducing local inflammation through the enhanced expression, phosphorylation, and nuclear translocation of SMAD2/3 (Levy et al., 2015; Cacheaux et al., 2009). Following TGF-β pathway activation, inward-rectifying potassium channels (Kir 4.1) in astrocytes, as well as glutamate transporter 1 and glutamate-aspartate transporter, are downregulated (Ivens et al., 2007; Löscher et al., 2020). Excitatory synaptogenesis increases (Weissberg et al., 2015), while perineuronal nets around GABAergic interneurons degrade (Kim et al., 2017) as a result of TGF-β signaling activation in astrocytes. These alterations disrupt the delicate balance between excitatory and inhibitory signals in the brain, leading to increased neuronal excitability and making neuronal circuits more prone to seizure generation. Epileptic activity, in turn, promotes gliosis and neuronal loss. However, perivascular PDGFRβ^+^ pericytes are elevated in conditions like focal cortical dysplasia (FCD) or temporal lobe epilepsy with hippocampal sclerosis (TLE-HS) (Garbelli et al., 2015). The pathogenic role of reduced vascular pericyte coverage in this animal model warrants further investigation.

The mechanism of epilepsy in pericyte knockout Cdk5 mice may also be linked to decreased polar distribution of AQP4. AQP4 exhibits significantly higher distribution at the astrocyte-pericyte interface compared to the astrocyte-endothelial cell interface (Gundersen et al., 2014; Hoddevik et al., 2017). Thus, reduced AQP4 polar distribution in pericyte knockout Cdk5 mice may relate to diminished pericyte vascular coverage. Perivascular AQP4 colocalizes with Kir4.1 (Smith and Verkman, 2015; Nagelhus et al., 2004), suggesting a functional partnership between these proteins. Astrocytic AQP4 cooperates with Kir4.1 in hippocampal function, and depletion of the perivascular AQP4 pool slows potassium (K^+^) clearance, potentially intensifying experimentally induced seizures (Eid et al., 2005; Amiry-Moghaddam et al., 2003). The observed reduction in AQP4 polar distribution in the brains of epileptic patients supports a plausible mechanism of epileptogenesis in this animal model (Eid et al., 2005; Peixoto-Santos et al., 2017).

The neurovascular unit plays a critical role in the development and progression of epilepsy (Reiss et al., 2023). While there are numerous animal models of epilepsy, such as pilocarpine, kainic acid, electrical or PTZ kindling models, etc. (Wang et al., 2022), there remains a scarcity of models specifically focused on cerebrovascular disease-related epilepsy (Liu et al., 2020). Our development of a mouse model of spontaneous epilepsy fills this gap, offering a valuable research tool to explore the role of the neurovascular unit in epilepsy onset and progression, investigate epilepsy pathogenesis, and evaluate potential therapies or interventions for epilepsy.

## Data Availability

The original contributions presented in this study are included in this article/supplementary material, further inquiries can be directed to the corresponding authors.
